# Plasma fibrinogen acts as a predictive factor for pathological complete response to neoadjuvant chemotherapy in breast cancer: a retrospective study of 1004 Chinese breast cancer patients

**DOI:** 10.1186/s12885-021-08284-8

**Published:** 2021-05-12

**Authors:** Yihua Wang, Yu Wang, Rui Chen, Zhenrong Tang, Yang Peng, Yudi Jin, Ailin Lan, Nan Ding, Yuran Dai, Linshan Jiang, Shengchun Liu

**Affiliations:** 1 Department of Endocrine and Breast Surgery, The First Affiliated Hospital of Chongqing Medical University, No.1 Youyi Road, Yuzhong District, Chongqing, 400016 China; 2 Department of Thyroid and Breast Surgery, The Affiliated Hospital of Zunyi Medical University, Zunyi, 563000 Guizhou Province China

**Keywords:** Breast cancer, Coagulation factor, Fibrinogen, Pathological complete response, Predictive ability

## Abstract

**Background:**

The aim of this study was to evaluate the relationship between pre-treatment plasma fibrinogen (Fib) level and pathological complete response (pCR) to neoadjuvant chemotherapy (NAC) in breast cancer patients and to assess the role of plasma Fib as a predictive factor.

**Methods:**

Data from 1004 consecutive patients with invasive breast cancer who received NAC and subsequent surgery were retrospectively analysed. Both univariate and multivariate analyses based on logistic regression model were performed to identify clinicopathological factors associated with pCR to NAC. Cox regression model was used to determine the correlation between clinical or pathological parameters and recurrence-free survival (RFS). The Kaplan-Meier method and the log-rank test were applied in the survival analysis.

**Results:**

The median value of Fib, rather than other plasma coagulation parameters, was significantly increased in non-pCR patients compared with pCR patients (*P* = 0.002). Based on the cut-off value estimated by the receiver operating characteristic (ROC) curve analysis, patients were divided into low or high Fib groups (Fib < 3.435 g/L or ≥ 3.435 g/L). Low Fib levels were significantly associated with premenopausal or perimenopausal status (*P* <  0.001), tumour size ≤5 cm (*P* = 0.002), and positive hormone receptor status (*P* = 0.002). After adjusted for other clinicopathological factors in the multivariate logistic regression model, low Fib status was strongly associated with pCR to NAC (OR = 3.038, 95% CI 1.667–5.537, *P* <  0.001). Survival analysis showed that patients with low Fib levels exhibited better 3-year RFS compared with patients with high Fib levels in the tumour size>5 cm group (77.5% vs 58.4%, log-rank, *P* = 0.0168).

**Conclusions:**

This study demonstrates that low pre-treatment plasma Fib (Fib < 3.435 g/L) is an independent predictive factor for pCR to NAC in breast cancer patients. Moreover, T3-featured breast cancer patients with lower Fib level exhibit better RFS outcomes after NAC compared with high Fib status.

**Supplementary Information:**

The online version contains supplementary material available at 10.1186/s12885-021-08284-8.

## Background

Breast cancer is currently the most common cancer (25% of all cancer cases) and is one of the leading causes of cancer-related death (15% of cancer deaths) among females worldwide [1]. Neoadjuvant chemotherapy (NAC) has become an integral part of the systematic treatment of breast cancer; NAC is used to convert unresectable to resectable cancers, to increase the rate of success for breast-conserving surgery and to evaluate the response to chemotherapy regimens before surgery [2]. It has been recognized that patients with a pathological complete response (pCR) after NAC have a significant survival advantage over those with residual invasive disease [3, 4].

Novel predictive biomarkers that can predict the pCR prior to NAC are valuable for making individualized treatment decisions and for maximizing efficacy in breast cancer patients. A series of studies have suggested an association between the haemostatic system and tumour biology [5, 6]. Different molecular mechanisms can cause the onset of a hypercoagulable state, and hypercoagulability in cancer patients has been implicated in angiogenesis, tumour cell invasion, tumour progression, and metastatic spread [6]. Subclinical hypercoagulable states have also been demonstrated in breast cancer patients [7, 8]. Fibrinogen (Fib), a key coagulation factor mainly synthesized by the liver, could be converted to fibrin by activated thrombin [9]. Previous studies have demonstrated that increased plasma Fib levels are frequently observed in cancer patients, and Fib has been shown to play a vital role in tumorigenesis and to contribute to angiogenesis, stroma formation, and hematogenous metastasis of tumour cells [10, 11].

Recent studies have shown that elevated pre-treatment plasma Fib levels are associated with poor prognosis in breast cancer [12–14]. However, there have been very few studies on the correlation between the pre-treatment plasma Fib level and the pCR to NAC in breast cancer patients. The aim of the present study was to evaluate the relationship between the pre-treatment plasma Fib level and the pCR to NAC in breast cancer patients and assess the role of Fib as a predictive factor.

## Methods

### Patients and treatments

**Fig. 1:**
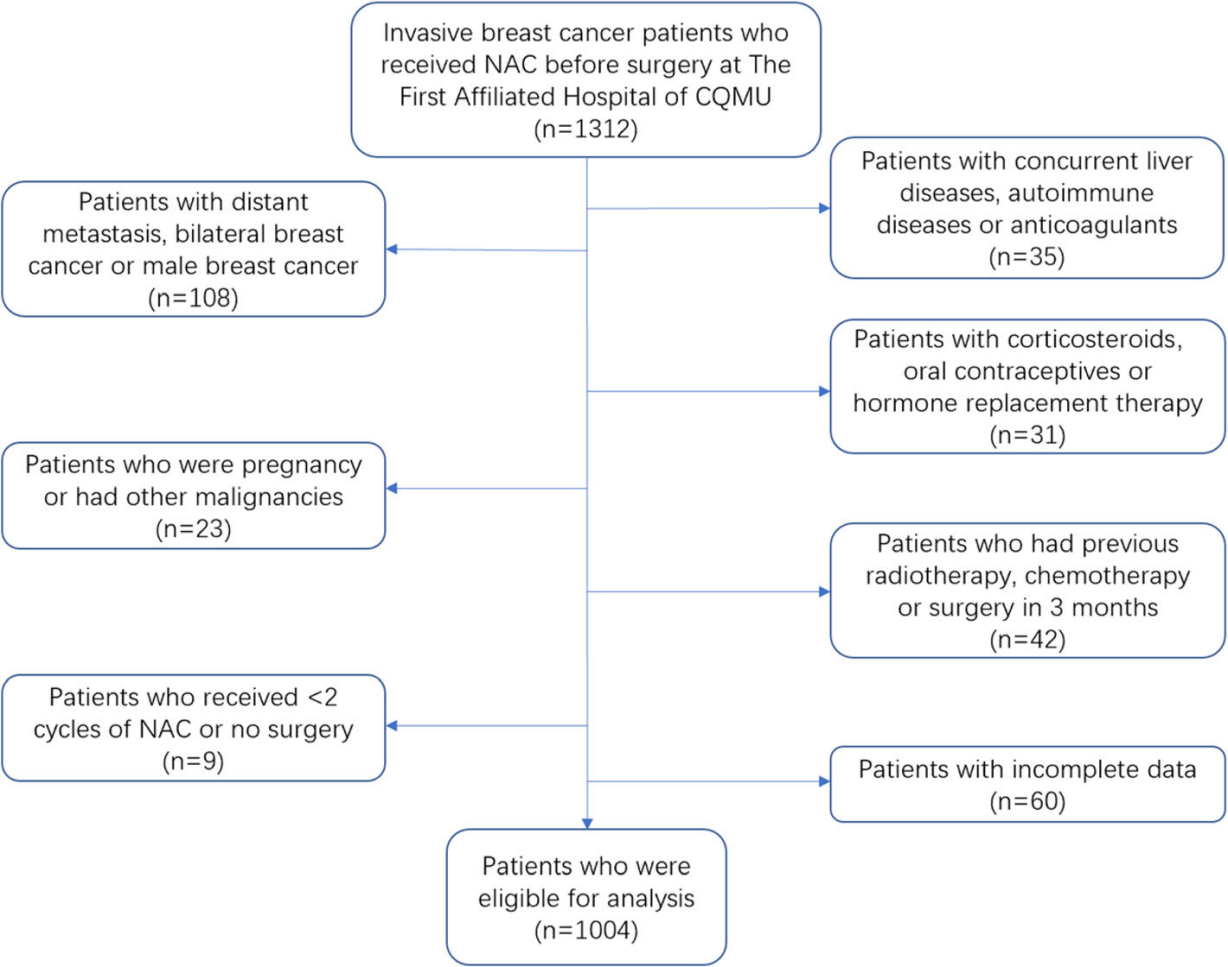
Flow chart of the patient selection. Abbreviations: NAC, neoadjuvant chemotherapy; CQMU, Chongqing Medical University

This retrospective study includes 1312 invasive breast cancer patients whose diagnoses were confirmed by histology and who received NAC from April 2012 to March 2019. The exclusion criteria for all participants were as follows: (1) patients with distant metastasis, bilateral breast cancer or male breast cancer, (2) patients with concurrent liver diseases, autoimmune diseases, haematological diseases or continuous anticoagulant treatment, (3) patients with corticosteroids, oral contraceptives or hormone replacement therapy, (4) patients who were pregnant or had previous cancer or concomitant cancer, (5) patients who had radiotherapy, chemotherapy or surgery in the previous 3 months, (6) patients who received<2 cycles of NAC or no surgery, or (7) patients with incomplete data. Ultimately, 1004 patients were eligible for analysis (Fig. 1). Medical records were reviewed to collect pre-treatment clinical data, such as age, menopausal status, tumour size, lymph node involvement, clinical stage (the 7th edition of the American Joint Committee on Cancer TNM Staging System) [15], histological subtype, oestrogen receptor (ER) status, progesterone receptor (PR) status, human epidermal growth factor receptor 2 (HER2) status, Ki67 index, and cycles of NAC. All patients received at least 2 but not more than 8 cycles of preoperative treatment with the standard anthracycline- and taxane-based regimen for breast cancer.

In our study, none of patients with HER2-positive status received neoadjuvant trastuzumab, though all of them were thoroughly informed of the effect of targeted therapy on the outcomes, due to the limitations of financial reasons and medical insurance in the neoadjuvant setting.

### Ethical statements

This research was conducted ethically in accordance with the World Medical Association Declaration of Helsinki and was approved by the Ethics Committee of The First Affiliated Hospital of Chongqing Medical University (ID: No. 2020–59), who deemed that written informed consent was not necessary due to the retrospective nature of the research and concealment of patient information.

### Blood sample analysis

Samples of 5 mL venous blood were collected in tubes with sodium citrate before NAC for the plasma coagulation test. The obtained blood samples were processed within 24 h to detect the plasma coagulation parameters, including Fib, prothrombin time (PT), prothrombin time ratio (PTR), international normalized ratio (INR), activated partial thromboplastin time (APTT), prothrombin activity (PTA), thrombin time (TT), fibrinogen degradation product (FDP), and D-dimer (DD) with a Sysmex CS5100 automatic coagulation analyser (Sysmex Corporation, Kobe, Japan) in centre laboratory of our hospital.

### Immunohistochemical staining and scoring

All breast cancer specimens were confirmed by core needle biopsy and tested using immunohistochemistry (IHC) to determine the tumour subtype. According to the 2011 St. Gallen consensus [16], the ER and PR status were considered positive if > 1% tumour cells were stained and the HER2 status was considered positive if > 10% of the tumour cells exhibited a 3+ score by IHC or a > 2.2-fold change compared to the expression of CEP17 in tumour cells via fluorescence in situ hybridization. Regarding Ki67, between 400 and 500 cells were counted to calculate the percentage of positive tumour cell nuclei, including hot spots, and 14% was defined as the optimal cut-off value. Hormone receptor (HR) positive was defined as ER and/or PR positive, while HR negative is defined as ER and PR negative. Based on HR status and HER2 status, the patients were classified according to the following subtypes: HR (+) HER2 (−), HR (+) HER2 (+), HR (−) HER2 (+), and HR (−) HER2 (−).

### Evaluation of chemotherapy response

A pCR was defined as the absence of residual invasive tumour lesions in any breast tissue and lymph node (ypT0ypN0 or ypT0/is ypN0) [17]. Participants were classified into the pCR group and the non-pCR group.

### Statistical methods

Data were analysed using SPSS (version 25.0) software (SPSS Inc., Chicago, IL, USA). The levels of plasma coagulation parameters were expressed as the median and interquartile range (IQR) because they are nonnormally distributed continuous variables, and the levels in the pCR group and the non-pCR group were compared by the Mann-Whitney U test and Wilcoxon signed-rank test. Receiver operating characteristic (ROC) curve analysis and the Youden index were used to calculate the optimal cut-off value for pre-treatment plasma Fib levels. Categorical variables were represented as numbers and percentages and compared via Chi-square and Fisher’s tests. Significant factors for pCR in univariate analyses were included in the multivariate logistic regression model with a forward LR method. The correlations between clinicopathological factors and recurrence-free survival (RFS) outcomes were investigated using univariate and multivariate analyses based on Cox regression model. Odds ratios (ORs) or hazard ratios (HRs) and corresponding 95% confidence intervals (CIs) with two-sided *P* values were used. To draw the survival rate curve of the included patients in different subgroups, the Kaplan-Meier method was applied. The log-rank test was used to determine the difference between survival curves. Statistical significance was defined as a two-sided *P* value < 0.05.

## Results

### Patient characteristics

A total of 1312 invasive breast cancer patients whose diagnoses were confirmed by histology and who received NAC before surgery in our institution were enrolled, and 1004 patients were eligible for analysis (Fig. 1). The baseline patient characteristics were shown in Table 1. The median age of all the patients was 49 years (IQR, 43–56 years). In total, 336 patients (33.5%) were premenopausal, 391 patients (38.9%) were perimenopausal, and 277 patients (27.6%) were postmenopausal. The most common tumour size was 2–5 cm (70.3%), followed by>5 cm (20.5%), and ≤ 2 cm (9.2%). The clinical lymph node involvement status was described as follows: N0 (36.4%), N1 (47.0%), and N2–3 (16.6%). The most of patients (69.9%) were categorized as being in stage II, according to the TNM staging system. Most patients (91.4%) received 4 chemotherapy cycles, and ductal (96.6%) was the most frequent histological subtype. The subtype distribution was as follows: 36.0% for HR (+) HER2 (−), 21.0% for HR (+) HER2 (+), 22.1% for HR (−) HER2 (+), 15.4% for HR (−) HER2 (−), and 5.5% for unknown. A total of 125 patients (12.5%) achieved pCR to NAC.

**Table 1:** Baseline characteristics of patients and tumours (*n* = 1004)

Characteristics	Number of cases (%)
**Age (years)**	
≤ 50	570 (56.8%)
> 50	434 (43.2%)
**Menstrual status**	
Premenopausal	336 (33.5%)
Perimenopausal	391 (38.9%)
Postmenopausal	277 (27.6%)
**Tumour size (cm)**	
≤ 2	92 (9.2%)
2–5	706 (70.3%)
> 5	206 (20.5%)
**Lymph node involvement**	
N0	365 (36.4%)
N1	472 (47.0%)
N2–3	167 (16.6%)
**Clinical stage**	
I	38 (3.8%)
II	702 (69.9%)
III	264 (26.3%)
**Histological subtype**	
Ductal	970 (96.6%)
Lobular	14 (1.4%)
Other	16 (1.6%)
Unknown	4 (0.4%)
**ER status**	
Positive	600 (59.8%)
Negative	404 (40.2%)
**PR status**	
Positive	441 (43.9%)
Negative	563 (56.1%)
**HER2 status**	
Positive	427 (42.5%)
Negative	522 (52.0%)
Unknown	55 (5.5%)
**Ki67 status (%)**	
≤ 14	266 (26.5%)
14–30	466 (46.4%)
> 30	272 (27.1%)
**Subtype**	
HR (+) HER2 (−)	361 (36.0%)
HR (+) HER2 (+)	211 (21.0%)
HR (−) HER2 (+)	222 (22.1%)
HR (−) HER2 (−)	155 (15.4%)
Unknown	55 (5.5%)
**Chemotherapy cycles**	
< 4	18 (1.8)
4	918 (91.4)
> 4	68 (6.8)
**Responder**	
pCR	125 (12.5)
cCR	73 (7.3)
PR	484 (48.2)
SD	301 (30.0)
PD	21 (2.0)

Abbreviations: *ER* oestrogen receptor, *PR* progesterone receptor, *HER2* human epidermal growth factor receptor 2, *HR* hormone receptor, *pCR* pathological complete response, *cCR* clinical complete response, *PR* partial response, *SD* stable disease, *PD* progressive disease

### Association between coagulation factors and pCR

**Fig. 2:**
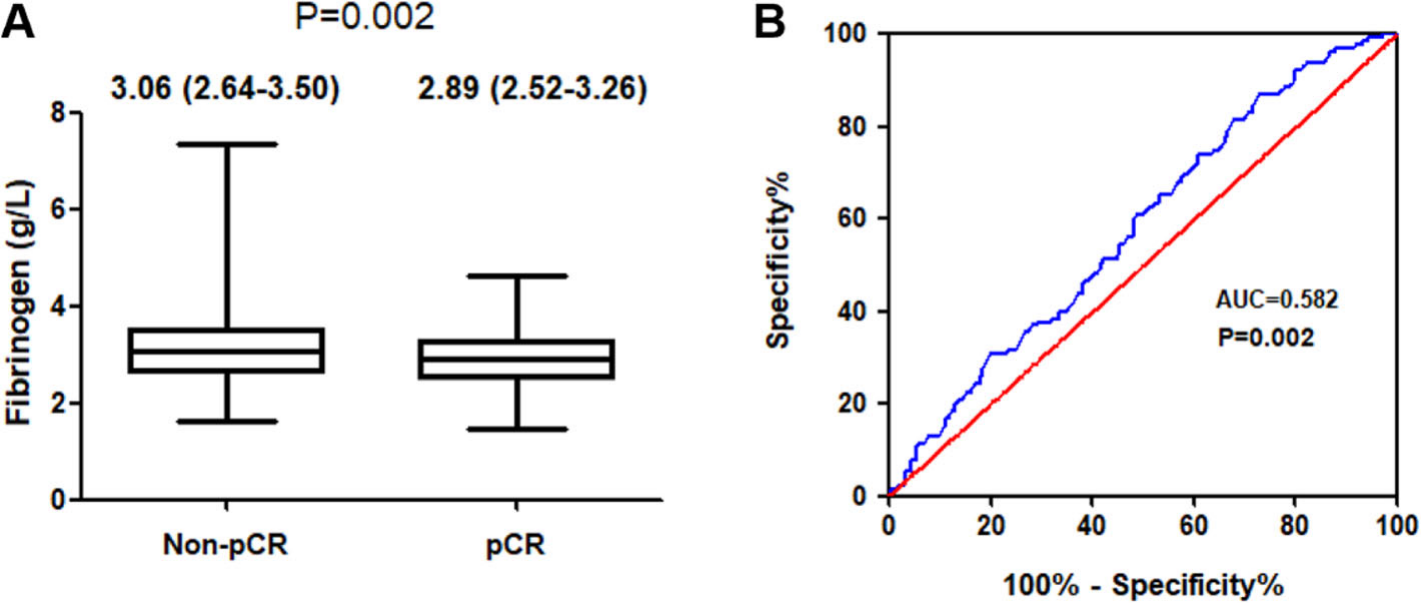
Critical analysis of Fib level for predicting pCR to NAC. Notes: **a** Plasma Fib levels, expressed as medians and interquartile ranges, were significantly increased among non-pCR patients compared with pCR patients. **b** ROC curve analysis was used to evaluate the optimal cutoff value of preoperative Fib levels for predicting the pCR in this study (*n* = 1004). The optimal Fib level cutoff value was identified according to the Youden index at 3.435 g/L. Abbreviations: Fib, fibrinogen; pCR, pathological complete response; NAC, neoadjuvant chemotherapy; ROC, receiver operating characteristics; AUC, area under curve

The plasma coagulation parameters were compared between the pCR group and the non-pCR group (Table 2). The median value of Fib was significantly increased in non-pCR patients compared with pCR patients (3.06 (2.64–3.50) g/L vs 2.89 (2.52–3.26) g/L, *P* = 0.002) (Fig. 2a). However, no significant differences in PT, PTR, INR, APTT, PTA, TT, FDP, and DD were noted between the pCR group and the non-pCR group (all *P* > 0.05, Table 2).

**Table 2:** Coagulation parameters comparison between pCR group and non-pCR group

	Median (*IQR*)			
Factors	Total (***n*** = 1004)	pCR (***n*** = 125)	Non-pCR (***n*** = 879)	***P*** value
**PT**	12.80 (12.40–13.30)	12.80 (12.40–13.20)	12.80 (12.40–13.30)	0.953
**PTR**	0.97 (0.94–1.01)	0.97 (0.94–1.01)	0.97 (0.94–1.01)	0.907
**INR**	0.96 (0.92–1.01)	0.96 (0.92–1.01)	0.96 (0.92–1.01)	0.785
**APTT**	35.40 (33.20–37.80)	35.50 (33.40–37.85)	35.40 (33.20–37.80)	0.704
**PTA**	107.00 (99.00–115.80)	108.00 (99.00–115.00)	107.00 (99.00–116.00)	0.857
**TT**	16.60 (16.00–17.20)	16.80 (16.30–17.30)	16.60 (16.00–17.20)	0.073
**Fib**	3.04 (2.63–3.45)	2.89 (2.52–3.26)	3.06 (2.64–3.50)	0.002
**FDP**	1.20 (0.80–1.60)	1.20 (0.85–1.60)	1.20 (0.725–1.60)	0.849
**DD**	0.27 (0.17–0.43)	0.28 (0.17–0.43)	0.26 (0.17–0.43)	0.975

Abbreviations: *IQR* interquartile range, *pCR* pathological complete response, *PT* prothrombin time, *PTR* prothrombin time ratio, *INR* international normalized ratio, *APTT* activated partial thromboplastin time, *PTA* prothrombin activity, *TT* thrombin time, *Fib* fibrinogen, *FDP* fibrinogen degradation product, *DD* D-dimer

### Relationship between Fib status and clinicopathological characteristics

ROC curve analysis and the Youden index were used to calculate the optimal cut-off value for Fib. Our results indicated that the optimal cut-off value for Fib was 3.435 g/L (*P* = 0.002) (Fig. 2b). Then, based on their low or high Fib status (Fib < 3.435 g/L and ≥ 3.435 g/L), the patients were divided into groups as shown in Table 3. The relationships between Fib status and clinicopathological characteristics in our study were assessed by the chi-square test. Our results indicated that the low Fib status was significantly associated with premenopausal or perimenopausal status (*P* <  0.001), tumour size ≤5 cm (*P* = 0.002), and positive HR status (*P* = 0.002). However, we failed to detect relationships between Fib status and other clinical characteristics, including age (*P* = 0.268), clinical lymph node involvement status (*P* = 0.943), histological subtype (*P* = 0.472), HER2 status (*P* = 0.275), Ki67 index > 14% (*P* = 0.052) and clinical responder (*P* = 0.053).

**Table 3:** Correlations between pre-treatment plasma fibrinogen status and clinicopathological features in breast cancer

	Fib status		
Characteristics	Low (***n*** = 744)	High (***n*** = 260)	***P*** value
**Age (years)**			0.268
≤ 50	430 (75.4%)	140 (24.6%)	
> 50	314 (72.4%)	120 (27.6%)	
**Menopausal status**			< 0.001
Pre/peri	563 (77.4%)	164 (22.6%)	
Post	181 (65.3%)	96 (34.7%)	
**Tumour size (cm)**			0.002
≤ 5	609 (76.3%)	189 (23.7%)	
> 5	135 (65.5%)	71 (34.5%)	
**Lymph node involvement**			0.943
No	270 (74.0%)	95 (26.0%)	
Yes	474 (74.2%)	165 (25.8%)	
**Histological subtype**			0.472
IDC	717 (73.9%)	253 (26.1%)	
Non-IDC	27 (79.4%)	7 (20.6%)	
**HR status**			0.002
Positive	475 (77.5%)	138 (22.5%)	
Negative	269 (68.8%)	122 (31.2%)	
**HER2 status**^**a**^			0.275
Positive	321 (75.2%)	106 (24.8%)	
Negative	376 (72.0%)	146 (28.0%)	
**Ki67 index (%)**			0.052
≤ 14	209 (78.6%)	57 (21.4%)	
> 14	535 (72.5%)	203 (27.5%)	
**Clinical responder**			0.053
CR (pCR + cCR)	162 (81.8%)	36 (18.2%)	
PR	349 (72.1%)	135 (27.9%)	
SD	218 (72.4%)	83 (27.6%)	
PD	15 (71.4%)	6 (28.6%)	

^a^55 cases with unknown HER2 status

Data are presented as number of cases (%)

Abbreviations: *Fib* fibrinogen, *IDC* invasive ductal carcinoma, *HR* hormone receptor, *HER2* human epidermal growth factor receptor 2, *CR* complete response, *pCR* pathological complete response, *cCR* clinical complete response, *PR* partial response, *SD* stable disease, *PD* progressive disease

### Relationship between the clinicopathological features and pCR

The chi-square test was used to assess the relationship between clinicopathological characteristics and pCR to NAC in breast cancer patients (Table 4). Clinicopathological factors were compared between the pCR group (*n* = 125) and the non-pCR group (*n* = 879). A higher rate of pCR after NAC was found in patients with tumour size ≤5 cm (*P* = 0.006), no lymph node involvement (*P* <  0.001), negative HR status (*P* <  0.001), molecular subtype as HR(−)HER2(+) or HR(−)HER2(−) (*P* <  0.001), Ki67 index > 14% (*P* <  0.001) and low Fib level (*P* <  0.001). However, no significant differences in pCR rates were found in age (*P* = 0.695), menopausal status (*P* = 0.241), histological subtype (*P* = 0.238), HER2 status (*P* = 0.215) and chemotherapy cycles (*P* = 0.689).

**Table 4:** The relationship between the clinicopathological features and pathological complete response after neoadjuvant chemotherapy in breast cancer

	Responder		
Characteristics	Non-pCR (***n*** = 879)	pCR (***n*** = 125)	***P*** value
**Age (years)**			0.695
≤ 50	497 (87.2%)	73 (12.8%)	
> 50	382 (88.0%)	52 (12.0%)	
**Menopausal status**			0.241
Pre/peri	631 (86.8%)	96 (13.2%)	
Post	248 (89.5%)	29 (10.5%)	
**Tumour size (cm)**			0.006
≤ 5	687 (86.1%)	111 (13.9%)	
> 5	192 (93.2%)	14 (6.8%)	
**Lymph node involvement**			< 0.001
No	291 (79.7%)	74 (20.3%)	
Yes	588 (92.0%)	51 (8.0%)	
**Histological subtype**			0.238
IDC	847 (87.3%)	123 (12.7%)	
Non-IDC	32 (94.1%)	2 (5.9%)	
**HR status**			< 0.001
Positive	561 (91.5%)	52 (8.5%)	
Negative	318 (81.3%)	73 (18.7%)	
**HER2 status**^**a**^			0.215
Positive	466 (89.3%)	56 (10.7%)	
Negative	370 (86.7%)	57 (13.3%)	
**Subtype**^**a**^			< 0.001
HR (+) HER2 (−)	338 (93.6%)	23 (6.4%)	
HR (+) HER2 (+)	187 (88.6%)	24 (11.4%)	
HR (−) HER2 (+)	187 (84.2%)	35 (15.8%)	
HR (−) HER2 (−)	124 (80.0%)	31 (20.0%)	
**Ki67 index (%)**			< 0.001
≤ 14	253 (95.1%)	13 (4.9%)	
> 14	626 (84.8%)	112 (15.2%)	
**Chemotherapy cycles**			0.689
< 4	65 (89.0%)	8 (11.0%)	
≥ 4	814 (87.4%)	117 (12.6%)	
**Fib status**			< 0.001
Low	635 (85.4%)	109 (14.6%)	
High	244 (93.9%)	16 (6.1%)	

^a^55 cases with unknown HER2 status and subtype

Data are presented as number of cases (%)

Abbreviations: *pCR* pathological complete response, *IDC* invasive ductal carcinoma, *HR* hormone receptor, *HER2* human epidermal growth factor receptor 2, *Fib* fibrinogen

### Ability of Fib to predict pCR to NAC

According to the Mann-Whitney U test and the chi-square test, the results indicated that PT, PTR, INR, APTT, PTA, TT, FDP, DD, age, menopausal status, histological subtype, HER2 status, and chemotherapy cycles were not significantly associated with pCR to NAC treatment (all *P*>0.05, Tables 2 and 4). Considering clinical practice and statistical power, TT, Fib status, tumour size, lymph node involvement, molecular subtype, Ki67 index, and chemotherapy cycles were included in the multivariate logistic regression analysis (Table 5). The results indicated that Fib status (*P* <  0.001), tumour size (*P* = 0.002), lymph node involvement (*P* <  0.001), molecular subtype (HR(−)HER2(+) vs HR (+) HER2(−), *P* = 0.003; HR(−)HER2(−) vs HR (+)HER2(−), *P* = 0.001) and Ki67 index (*P* = 0.001) were the potential factors affecting the pCR to NAC. After adjusting for other factors in the logistic regression model, Fib was demonstrated to be an independent predictive factor for pCR to NAC, and low Fib level (Fib < 3.435 g/L) was strongly associated with a better pCR rate (OR = 3.038, 95% CI 1.667–5.537, *P* <  0.001) (Table 5). Although a considerable proportion of included patients were HR (+), we found that Fib was an independent predictor of pCR both in the total population and the two subgroups stratified by HR status of this study (Table 5, Tables S1 and S2).

**Table 5:** Logistic regression analysis of clinicopathological factors and pathological complete response after neoadjuvant chemotherapy in breast cancer

Factors		Multivariate analysis	
	OR	95% CI	***P*** value
**TT (continuous)**	–	–	0.881
**Fib status (low vs high)**	3.038	1.667–5.537	< 0.001
**Tumour size (≤ 5 cm vs > 5 cm)**	3.052	1.484–6.276	0.002
**Lymph node involvement (no vs yes)**	2.840	1.863–4.328	< 0.001
**Subtype**			
**HR (+) HER2 (−)**	1	Ref.	Ref.
**HR (+) HER2 (+)**	1.594	0.858–2.961	0.140
**HR (−) HER2 (+)**	2.410	1.348–4.307	0.003
**HR (−) HER2 (−)**	2.954	1.597–5.464	0.001
**Ki67 index (> 14% vs ≤ 14%)**	3.163	1.633–6.125	0.001
**Chemotherapy cycles (≥ 4 vs < 4)**	**–**	–	0.679

Abbreviations: *CI* confidence interval, *OR* odds ratio, *TT* thrombin time, *Fib* fibrinogen, *HR* hormone receptor, *HER2* human epidermal growth factor receptor 2

### RFS outcomes of the included patients and the association with Fib status

As of 1 Feb, 2020, we have conducted follow-up among totally 1004 patients (the loss rate of follow-up was 14.6%). As for the total population (*n* = 1004), the median RFS was 49 months (95%CI 46.64–51.36). For patients with potential follow-up of more than 3 years (*n* = 826), the median RFS was 55 months (95%CI 52.43–57.57).

**Fig. 3:**
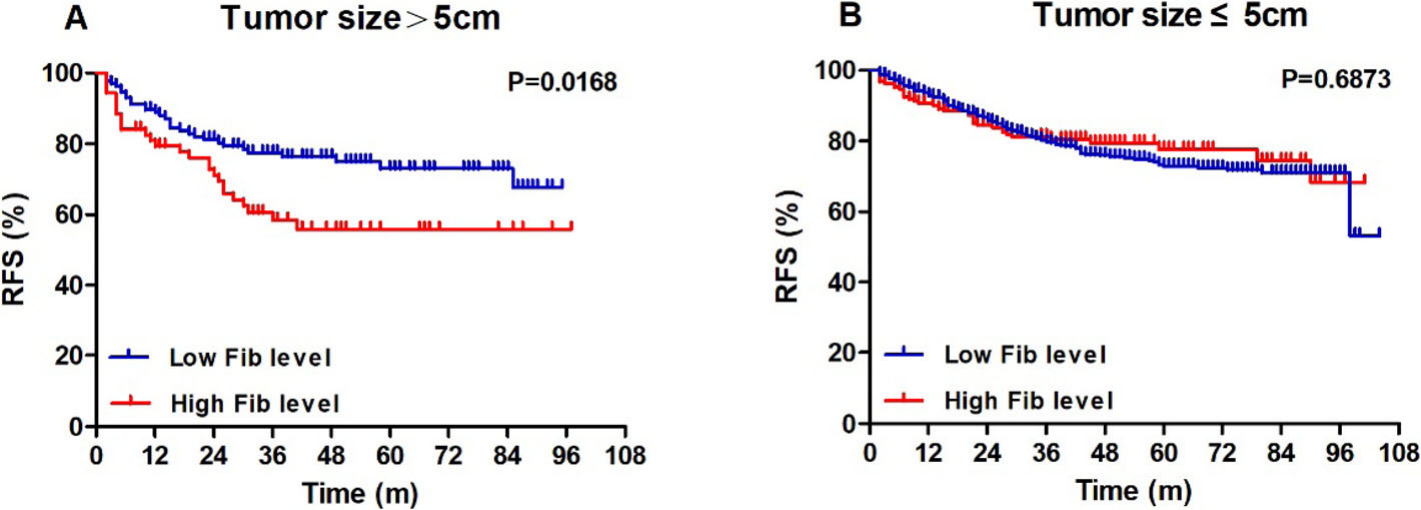
RFS outcomes in the tumor>5 cm and tumor ≤5 cm groups by Fib status (all patients, *n* = 1004). Notes: **a** In the tumor size>5 cm group, patients with low Fib levels exhibited better 3-year RFS compared with patients with high Fib levels (77.5% vs 58.4%, log-rank, *P* = 0.0168). **b** In the tumor size ≤5 cm group, no significant differences were showed in RFS graphs (log-rank, *P* = 0.6873). Abbreviations: RFS, recurrence-free survival; Fib, fibrinogen

**Fig. 4:**
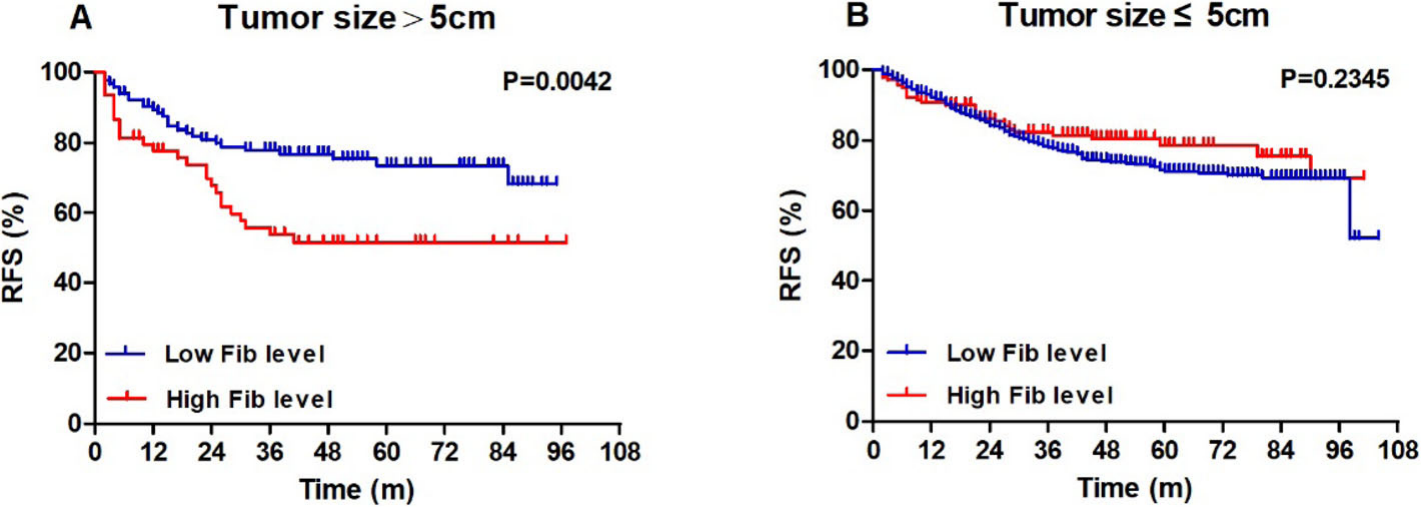
RFS outcomes in the tumor>5 cm and tumor ≤5 cm groups by Fib status (patients with potential follow-up of more than 3 years, *n* = 826). Notes: **a** In the tumor size>5 cm group, patients with low Fib levels exhibited better 3-year RFS compared with patients with high Fib levels (77.9% vs 53.8%, log-rank, *P* = 0.0042). **b** In the tumor size ≤5 cm group, no significant differences were showed in RFS graphs (log-rank, *P* = 0.2345). Abbreviations: RFS, recurrence-free survival; Fib, fibrinogen

Among the retrieved clinicopathological factors (including Fib status) of all patients, invasive ductal histology (HR = 2.093), tumour size > 5 cm (HR = 1.452), lymph node involvement (HR = 1.742), specific molecular subtypes (HR*(+)HER2(+) vs HR*(+)HER2(−), HR = 1.529; HR*(−)HER2(+) vs HR*(+)HER2(−), HR = 2.195; HR*(−)HER2(−) vs HR*(+)HER2(−), HR = 2.843) and non-pCR (HR = 1.794) were associated with worse RFS outcomes (all *P* <  0.05, Table 6). For those 826 patients with potential follow-up of more than 3 years, tumour size > 5 cm, lymph node involvement, HR*(−)HER2(+), HR*(−)HER2(−) and non-pCR were associated with unfavourable RFS (HR > 1, *P* <  0.05, Table 7). Notably, Fib status and all other coagulation parameters were not correlated with the RFS outcomes of the total population and those with more than 3-year follow-up time (*P* > 0.05, Tables 6, 7 and S3). When stratified by tumour size (>5 cm or ≤ 5 cm), as shown in Fig. 3, patients with low Fib levels exhibited better 3-year RFS compared with patients with high Fib levels in the tumour size>5 cm group (77.5% vs 58.4%, log-rank, *P* = 0.0168). However, in the tumour size ≤5 cm group, no significant differences were showed in RFS graphs (log-rank, *P* = 0.6873). Similar results were found among patients with potential follow-up of more than 3 years (Fig. 4). Additionally, after stratified by HR status, the survival analysis plotted as Figure S1 showed that only HR-negative patients with pCR exhibited better 3-year RFS compared with non-pCR patients (80.1% vs 67.4%, log-rank, *P* = 0.0264). This finding was not true of the HR-positive group (*P* = 0.2312).

**Table 6:** Univariate and multivariate analyses for RFS in all patients (*n* = 1004)

	RFS					
	Univariate analysis			Multivariate analysis		
Factors	HR	95%CI	***P*** value	HR	95%CI	***P*** value
**Age (> 50y vs ≤ 50y)**	0.831	0.632–1.093	0.186	–	–	–
**Menopausal status (pre/peri vs post)**	1.072	0.793–1.450	0.649	–	–	–
**Histological subtype (non-IDC vs IDC)**	1.922	1.080–3.419	0.026	2.093	1.184–3.701	0.011
**Tumour size (> 5 cm vs ≤ 5 cm)**	1.454	1.070–1.976	0.017	1.452	1.073–1.965	0.016
**Lymph node involvement (yes vs no)**	1.720	1.266–2.338	0.001	1.742	1.282–2.366	< 0.001
**Subtype**						
HR*(+) HER2 (−)	1	Ref.	Ref.	1	Ref.	Ref.
HR*(+) HER2 (+)	1.395	0.945–2.059	0.094	1.529	1.033–2.264	0.034
HR*(−) HER2 (+)	1.930	1.359–2.743	< 0.001	2.195	1.541–3.128	< 0.001
HR*(−) HER2 (−)	2.430	1.673–3.530	< 0.001	2.843	1.948–4.148	< 0.001
**Ki67 index (≤ 14% vs > 14%)**	1.271	0.911–1.773	0.157	–	–	0.215
**Fib status (high vs low)**	1.024	0.754–1.391	0.880	–	–	0.951
**pCR (no vs yes)**	1.851	1.612–5.487	0.025	1.794	1.051–3.063	0.032

Abbreviations: *RFS* recurrence-free survival, *HR* hazard ratio, *CI* confidence interval, *IDC* invasive ductal carcinoma, HR* hormone receptor, *HER2* human epidermal growth factor receptor 2, *Fib* fibrinogen, *pCR* pathological complete response

**Table 7:** Univariate and multivariate analyses for RFS in patients with potential follow-up of more than 3 years (*n* = 826)

	RFS					
	Univariate analysis			Multivariate analysis		
Factors	HR	95% CI	***P*** value	HR	95% CI	***P*** value
**Age (> 50y vs ≤ 50y)**	0.873	0.653–1.166	0.356	–	–	–
**Menopausal status (pre/peri vs post)**	1.044	0.757–1.440	0.793	–	–	–
**Histological subtype (non-IDC vs IDC)**	1.608	0.837–3.092	0.154	–	–	0.086
**Tumour size (> 5 cm vs ≤ 5 cm)**	1.407	1.022–1.939	0.036	1.433	1.046–1.965	0.025
**Lymph node involvement (yes vs no)**	1.763	1.281–2.427	0.001	1.727	1.258–2.372	0.001
**Subtype**						
HR* (+) HER2 (−)	1	Ref.	Ref.	1	Ref.	Ref.
HR* (+) HER2 (+)	1.350	0.890–2.046	0.158	1.409	0.929–2.136	0.107
HR* (−) HER2 (+)	1.935	1.338–2.800	< 0.001	2.201	1.517–3.192	< 0.001
HR* (−) HER2 (−)	2.391	1.626–3.516	< 0.001	2.746	1.861–4.051	< 0.001
**Ki67 index (≤ 14% vs > 14%)**	1.325	0.936–1.874	0.112	–	–	0.163
**Fib status (high vs low)**	1.095	0.787–1.523	0.589	–	–	0.584
**pCR (no vs yes)**	1.945	1.090–3.471	0.024	1.903	1.072–3.380	0.028

Abbreviations: *RFS* recurrence-free survival, *HR* hazard ratio, *CI* confidence interval, *IDC* invasive ductal carcinoma, HR* hormone receptor, *HER2* human epidermal growth factor receptor 2, *Fib* fibrinogen, *pCR* pathological complete response

## Discussion

Nowadays, NAC has become an integral part of the systematic treatment of breast cancer [1]. A major advantage of this strategy is the ability to observe the tumour response to chemotherapy regimens before surgery [2]. It is well known that the prognosis of patients with locally advanced breast cancer is closely related to whether pCR is achieved after NAC. Patients with pCR after NAC could have a better prognosis than those without [3, 4]. In this study, we examined 1004 consecutive breast cancer patients who received NAC to provide evidence for the predictive value of Fib in pCR. The main finding of our analysis is that a lower pre-treatment Fib level is a significant independent predictor of pCR (OR = 3.038, 95% CI 1.667–5.537, *P* <  0.001) and was associated with premenopausal or perimenopausal status (*P* <  0.001), tumour size ≤5 cm (*P* = 0.002), and positive HR status (*P* = 0.002) in breast cancer patients. Moreover, after stratified by tumour size, the survival analysis showed that patients with low Fib levels exhibited better 3-year RFS compared with patients with high Fib levels in the tumour size>5 cm group (77.5% vs 58.4%, log-rank, *P* = 0.0168; Fig. 3). But in the tumour size ≤5 cm group, no significant differences were showed in RFS graphs (log-rank, *P* = 0.6873; Fig. 3). After excluding those patients with potential less than 3-year follow-up, the survival analysis showed similar results (Fig. 4).

Fib is a 340 kDa glycoprotein that is mainly synthesized in the liver upon inflammatory stimulation by IL-6 and IL-1β [9]. It is generally regarded as a key factor of coagulation and fibrinolytic activation. Recently, a series of studies have also reported the prognostic and predictive value of coagulation factors especially the change in the Fib levels across different cancer types [12–14, 18–25], including breast cancer [12–14, 22, 25]. In a large retrospective study of 2073 consecutive breast cancer patients, Wen et al. [12] reported that an elevated preoperative plasma Fib level was an independent prognostic factor for overall survival in breast cancer patients who underwent surgical treatment (*P* = 0.001). Additionally, a retrospective analysis including 520 consecutive breast cancer patients found that an increased pre-treatment plasma Fib level was closely associated with shorter disease-specific survival (*P* = 0.042) [13].

Previous studies have demonstrated that Fib can also be endogenously synthesized by breast cancer cells themselves [6, 26]. Several mechanisms can explain the contribution of Fib to tumour cell infiltration and expansion. First, due to the strong procoagulant effect of tumour cells, a large amount of Fib is aggregated around tumour cells and converted into fibrin, which is involved in metastasis and new vessel formation and promotes the formation of tumour stromal tissue [26, 27]. Second, Shu et al. demonstrated that high concentrations of Fib can induce epithelial-mesenchymal transition [28], which confers migration, invasion and metastasis capacities to tumour cells and renders tumour cells resistant to multiple drugs [29]. Moreover, Fib can promote tumour proliferation and stimulate angiogenesis through interactions with fibroblast growth factor 2 and vascular endothelial growth factor [9, 30]. In addition, Fib can act as a bridge between the tumour and the host cell and support the adhesion of tumour cells to the vascular endothelium of target organs [30, 31].

Few studies have reported the relationship between the pre-treatment Fib level and pCR to NAC in breast cancer. In a study including 67 breast cancer patients receiving NAC, Mei et al. revealed that patients who had decreased Fib levels after NAC had a better clinical response than patients who had stable or increased Fib levels [32]. In our centre, implantable venous access port systems or peripherally inserted central venous catheters are routinely provided to breast cancer patients before chemotherapy, which may promote the hypercoagulable state of the blood [33]. The comparison of plasma Fib levels before and after chemotherapy may be unreliable. Therefore, we focused on the relationship between the pre-treatment plasm Fib status and the pCR of NAC in breast cancer patients.

In our study, the median value of Fib was significantly increased in pCR patients compared with non-pCR patients (3.06 (IQR 2.64–3.50) g/L vs 2.89 (IQR 2.52–3.26) g/L, *P* = 0.002), while no significant differences in PT, PTR, INR, APTT, PTA, FDP, and DD were noted between the pCR group and the non-pCR group. Some studies suggest that DD may be related to the clinical stage and prognosis of breast cancer [7, 34]. But we failed to observe the relationship between DD and pCR to NAC in breast patients. According to ROC curve analysis and the Youden index, the optimal cut-off value was 3.435 g/L (*P* = 0.002). This value is discrepant with those previously reported [12–14] due to the differences in sample sources and predicted targets. A higher pCR rate was noted in the low Fib group (a value less than 3.435 g/L) (*P* <  0.001). Adjusted for other clinicopathological factors in the logistic regression model, low Fib status was still strongly associated with a better pCR rate (OR = 3.038, 95% CI 1.667–5.537, *P* <  0.001). However, in 84 oesophageal cancer patients, higher pre-treatment plasma Fib levels were found to be significantly associated with a better histological response to neoadjuvant treatment [24]. This finding seems to contradict our results. This may be due to differences in sample size and tumour nature. Moreover, our results showed that lower plasma Fib levels were significantly associated with premenopausal or perimenopausal status (*P* <  0.001), tumour size ≤5 cm (*P* = 0.002), and positive HR status (*P* = 0.002). These are similar to published studies about Fib and clinicopathological factors in breast cancer [12–14].

There were some limitations to this study. First, due to the retrospective nature of the current study, a selection bias is unavoidable. To limit interference factors, some exclusion criteria were set, but the relationship between these factors and breast cancer was not evaluated. Second, most patients (91.4%) received NAC for 4 cycles, and even a small number of patients (1.8%) received less than 4 cycles of NAC. This chemotherapy regimen could be considered as suboptimal. And all included HER2-positive patients did not receive anti-HER2 therapy with NAC due to financial reasons and medical insurance, which did not cover the anti-HER2 drugs such as trastuzumab in the neoadjuvant setting, so the pCR rates of this cohort were lower than those reported in other literature [35]. Such a low pCR rate in this cohort may confound the findings of the predictive value of fibrinogen level. Third, the absence of the data on the Residual Cancer Burden (RCB) system may be another limitation in this study. The RCB system is not a routine strategy for the assessment of pathological response by the pathological diagnosis institution in our centre. However, this study may offer more evidence and recognition for the relationship between Fib and breast cancer and facilitate the administration of NAC therapy to achieve a better pCR rate. Notably, survival analysis showed that lower Fib levels were correlated with better 3-year RFS in the larger tumour size (>5 cm) group which is another merit of our study. Still, further prospective trials are needed to confirm the predictive significance of Fib in breast cancer.

## Conclusions

This study demonstrates that Fib was significantly associated with menopausal status, tumour size, ER status, and PR status. In addition, preoperative low plasma Fib status (Fib < 3.435 g/L) is an independent predictive factor for pCR to NAC in breast cancer patients. More importantly, lower Fib levels were correlated with better 3-year RFS in the larger tumour size (>5 cm) group. Breast cancer patients with low pre-treatment Fib levels should be recommended to receive NAC in order to achieve pCR and obtain clinical benefits.

## Supplementary Information

**Additional file 1: Table S1.** Logistic regression analysis of clinicopathological factors and pathological complete response after neoadjuvant chemotherapy in HR (+) breast cancer.

**Additional file 2: Table S2.** Logistic regression analysis of clinicopathological factors and pathological complete response after neoadjuvant chemotherapy in HR (−) breast cancer.

**Additional file 3: Table S3.** Cox regression analysis of coagulation parameters and RFS in breast cancer.

**Additional file 4: Figure S1.** RFS outcomes in the pCR and non-pCR groups by HR status.

**Additional file 5: Supplementary material 1.** RFS outcomes by molecular subtypes. **Supplementary material 2.** Survival analysis on Fib levels and RFS by molecular subtypes. **Supplementary material 3.** Survival analysis on Fib levels and RFS by molecular subtypes (potential survival more than 3 years).

## Data Availability

The data used and analysed during the current study are available from the corresponding author on reasonable request.

## Abbreviations

NAC: Neoadjuvant chemotherapy
pCR: Pathological complete response
Fib: Fibrinogen
TNM: Tumour-node-metastasis
ER: Oestrogen receptor
PR: Progesterone receptor
HER2: Human epidermal growth factor receptor 2
PT: Prothrombin time
PTR: Prothrombin time ratio
INR: International normalized ratio
APTT: Activated partial thromboplastin time
PTA: Prothrombin activity
TT: Thrombin time
FDP: Fibrinogen degradation product
DD: D-dimer
IHC: Immunohistochemistry
HR: Hormone receptor
IQR: Interquartile range
ROC: Receiver operating characteristic
RFS: Recurrence-free survival
ORs: Odds ratios
HRs: Hazard ratios
CIs: Confidence intervals
RCB: Residual cancer burden
