# Supplementary material for: Plasma fibrinogen acts as a predictive factor for pathological complete response to neoadjuvant chemotherapy in breast cancer: a retrospective study of 1004 Chinese breast cancer patients
Source: BMC Cancer. 2021 May 12;21:542. doi: 10.1186/s12885-021-08284-8 (PMC8114717; doi:10.1186/s12885-021-08284-8)
Supplement: Supplementary file 1 — Additional file 1: Table S1. Logistic regression analysis of clinicopathological factors and pathological complete response after neoadjuvant chemotherapy in HR (+) breast cancer. [file 12885_2021_8284_MOESM1_ESM.docx]

**Table S1** Logistic regression analysis of clinicopathological factors and pathological complete response after neoadjuvant chemotherapy in HR (+) breast cancer

| **Factors** |  | **Multivariate analysis** |  |
| --- | --- | --- | --- |
|  | **OR** | **95% CI** | ***P* value** |
| **TT (continuous)** | **-** | - | 0.565 |
| **Fib status (low *vs* high)** | 3.800 | 1.311-11.010 | 0.014 |
| **Tumor size (≤ 5cm *vs* > 5 cm)** | 3.352 | 1.002-11.209 | 0.050 |
| **Lymph node involvement (no *vs* yes)** | 3.110 | 1.662-5.819 | < 0.001 |
| **HER2 status (positive *vs* negative)** | **-** | - | 0.149 |
| **Ki67 index (> 14% *vs* ≤ 14%)** | 5.020 | 1.920-13.125 | 0.001 |
| **Chemotherapy cycles (≥ 4 *vs* < 4)** | **-** | - | 0.252 |

**Abbreviations:** HR, hormone receptor; OR, odds ratio; CI, confidence interval; TT, thrombin time; Fib, fibrinogen; HER2, human epidermal growth factor receptor 2.
