# Supplementary material for: Plasma fibrinogen acts as a predictive factor for pathological complete response to neoadjuvant chemotherapy in breast cancer: a retrospective study of 1004 Chinese breast cancer patients
Source: BMC Cancer. 2021 May 12;21:542. doi: 10.1186/s12885-021-08284-8 (PMC8114717; doi:10.1186/s12885-021-08284-8)
Supplement: Supplementary file 2 — Additional file 2: Table S2. Logistic regression analysis of clinicopathological factors and pathological complete response after neoadjuvant chemotherapy in HR (−) breast cancer. [file 12885_2021_8284_MOESM2_ESM.docx]

**Table S2** Logistic regression analysis of clinicopathological factors and pathological complete response after neoadjuvant chemotherapy in HR (-) breast cancer

| **Factors** |  | **Multivariate analysis** |  |
| --- | --- | --- | --- |
|  | **OR** | **95% CI** | ***P* value** |
| **TT (continuous)** | **-** | - | 0.376 |
| **Fib status (low *vs* high)** | 2.642 | 1.274-5.477 | 0.009 |
| **Tumor size (≤ 5cm *vs* > 5 cm)** | 2.919 | 1.185-7.188 | 0.020 |
| **Lymph node involvement (no *vs* yes)** | 2.995 | 1.708-5.250 | < 0.001 |
| **HER2 status (positive *vs* negative)** | **-** | - | 0.250 |
| **Ki67 index (> 14% *vs* ≤ 14%)** | **-** | - | 0.120 |
| **Chemotherapy cycles (≥ 4 *vs* < 4)** | **-** | - | 0.372 |

**Abbreviations:** HR, hormone receptor; OR, odds ratio; CI, confidence interval; TT, thrombin time; Fib, fibrinogen; HER2, human epidermal growth factor receptor 2.
