# Supplementary material for: Plasma fibrinogen acts as a predictive factor for pathological complete response to neoadjuvant chemotherapy in breast cancer: a retrospective study of 1004 Chinese breast cancer patients
Source: BMC Cancer. 2021 May 12;21:542. doi: 10.1186/s12885-021-08284-8 (PMC8114717; doi:10.1186/s12885-021-08284-8)
Supplement: Supplementary file 3 — Additional file 3: Table S3. Cox regression analysis of coagulation parameters and RFS in breast cancer. [file 12885_2021_8284_MOESM3_ESM.docx]

**Table S3** Cox regression analysis of coagulation parameters and RFS in breast cancer

| **Factors** |  | **Multivariate analysis** |  |
| --- | --- | --- | --- |
|  | **HR** | **95% CI** | ***P* value** |
| **PT (continuous)** | **-** | - | 0.510 |
| **PTR (continuous)** | **-** | - | 0.188 |
| **INR (continuous)** | **-** | - | 0.745 |
| **APTT (continuous)** | **-** | - | 0.306 |
| **PTA (continuous)** | **-** | - | 0.394 |
| **TT (continuous)** | **-** | - | 0.367 |
| **Fib (continuous)** | **-** | - | 0.681 |
| **FDP (continuous)** | **-** | - | 0.799 |
| **DD (continuous)** | **-** | - | 0.604 |

**Abbreviations:** RFS, recurrence-free survival; PT, prothrombin time; PTR, prothrombin time ratio; INR, international normalized ratio; APTT, activated partial thromboplastin time; PTA, prothrombin activity; TT, thrombin time; Fib, fibrinogen; FDP, fibrinogen degradation product; DD, D-dimer.
