# Supplementary material for: Plasma fibrinogen acts as a predictive factor for pathological complete response to neoadjuvant chemotherapy in breast cancer: a retrospective study of 1004 Chinese breast cancer patients
Source: BMC Cancer. 2021 May 12;21:542. doi: 10.1186/s12885-021-08284-8 (PMC8114717; doi:10.1186/s12885-021-08284-8)
Supplement: Supplementary file 4 — Additional file 4: Figure S1. RFS outcomes in the pCR and non-pCR groups by HR status. [file 12885_2021_8284_MOESM4_ESM.docx]

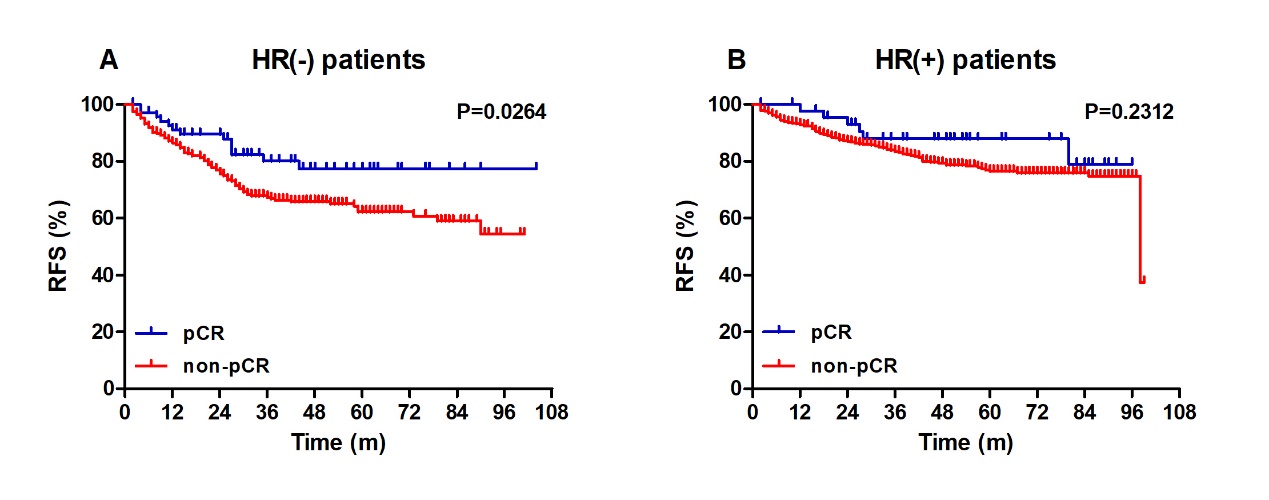


**Figure S1** RFS outcomes in the pCR and non-pCR groups by HR status

**Notes:** (**A**) In the HR (-) group, patients with pCR exhibited better 3-year RFS compared with non-pCR patients (80.1% *vs* 67.4%, log-rank, *P* = 0.0264). (**B**) In the HR (+) group, no significant differences were showed in RFS graphs (log-rank, *P* = 0.2312).

**Abbreviations:** RFS, recurrence-free survival; pCR, pathological complete response; HR, hormone receptor.
