# Supplementary material for: Plasma fibrinogen acts as a predictive factor for pathological complete response to neoadjuvant chemotherapy in breast cancer: a retrospective study of 1004 Chinese breast cancer patients
Source: BMC Cancer. 2021 May 12;21:542. doi: 10.1186/s12885-021-08284-8 (PMC8114717; doi:10.1186/s12885-021-08284-8)
Supplement: Supplementary file 5 — Additional file 5: Supplementary material 1. RFS outcomes by molecular subtypes. Supplementary material 2. Survival analysis on Fib levels and RFS by molecular subtypes. Supplementary material 3. Survival analysis on Fib levels and RFS by molecular subtypes (potential survival more than 3 years). [file 12885_2021_8284_MOESM5_ESM.docx]

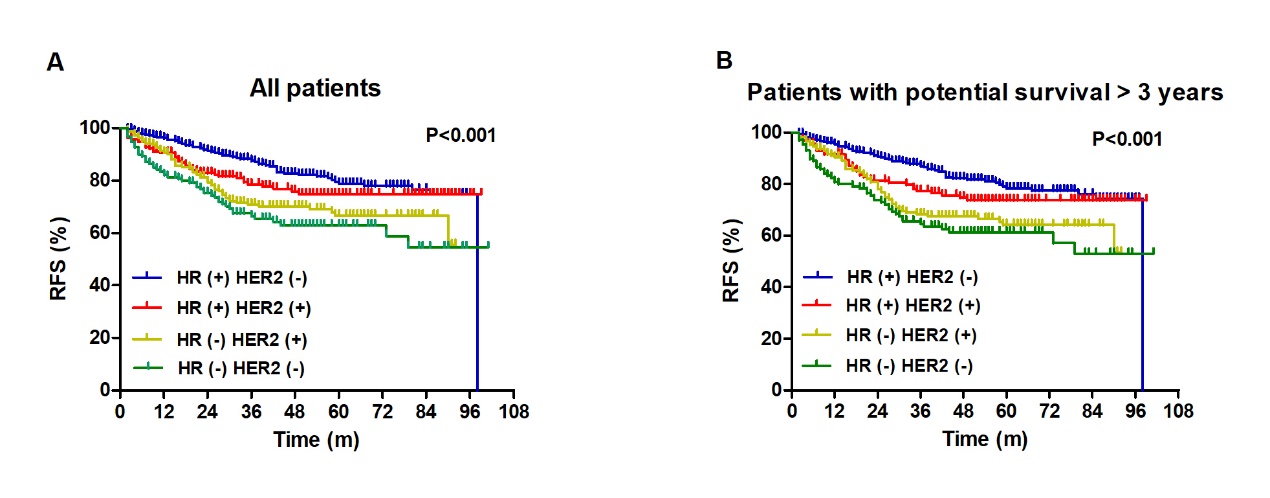
**Supplementary material 1**. RFS outcomes by molecular subtypes


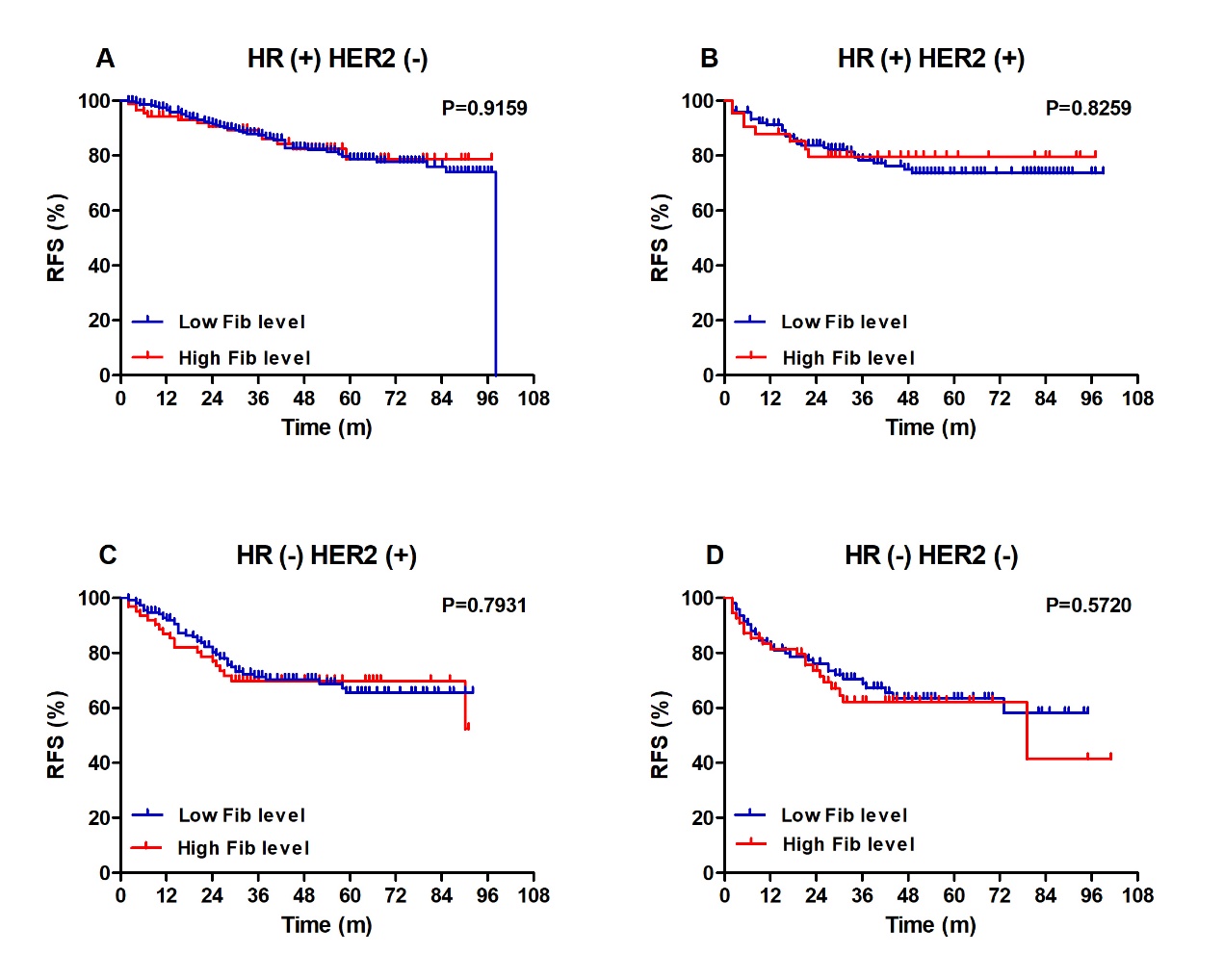
**Supplementary material 2**. Survival analysis on Fib levels and RFS by molecular subtypes


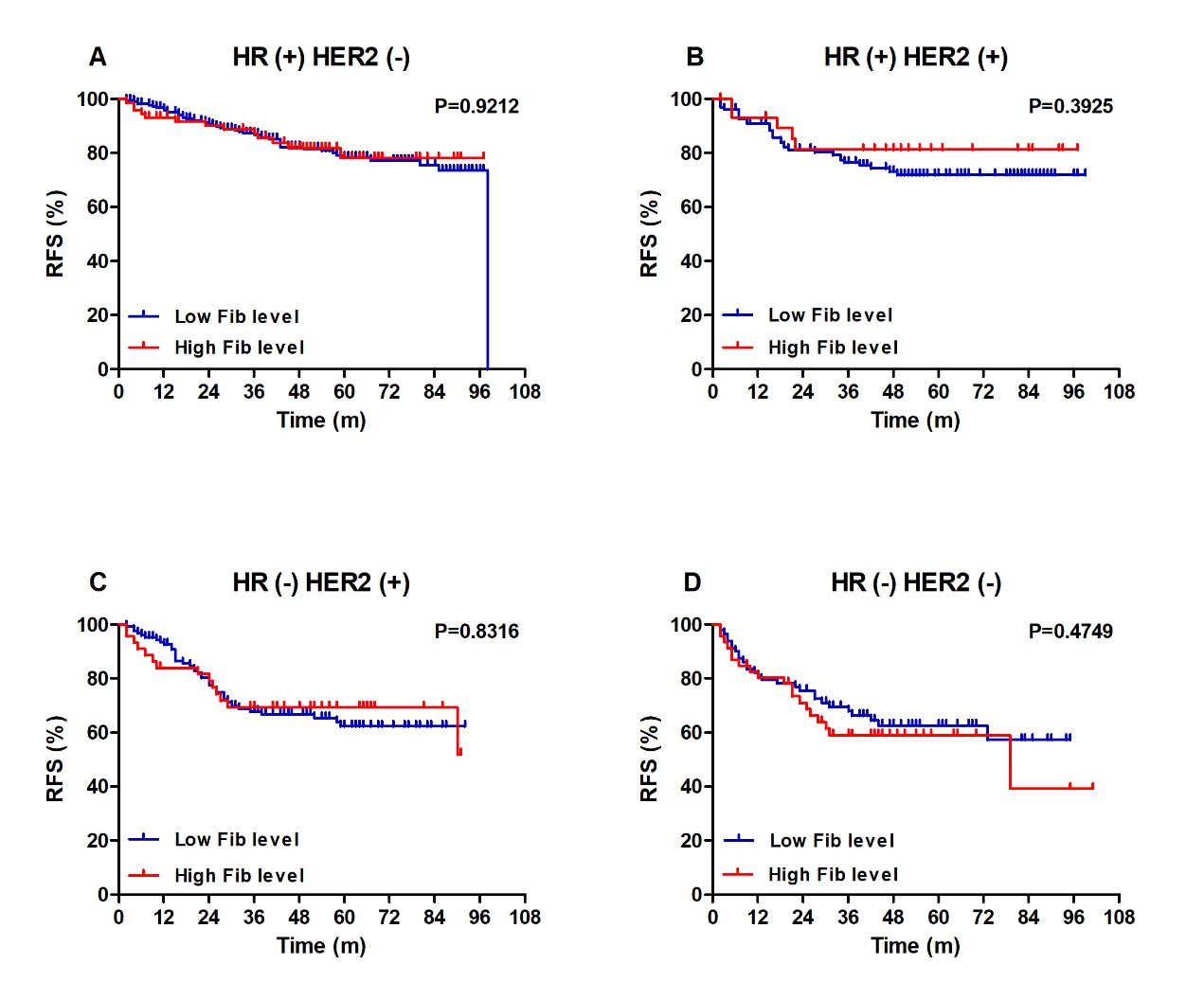
**Supplementary material 3**. Survival analysis on Fib levels and RFS by molecular subtypes (potential survival more than 3 years)
